# Temperature Trajectories Are Associated With the Prognosis of Septic Patients

**DOI:** 10.1155/emmi/9111659

**Published:** 2026-05-30

**Authors:** Keying Ding, Chang Xu, Xueqi Zhu, Hua Wang, Zhaojun Xu, Zhongqiu Lu

**Affiliations:** ^1^ Emergency Department, The First Affiliated Hospital of Wenzhou Medical University, Wenzhou, 325000, China, wzhospital.cn; ^2^ Wenzhou Key Laboratory of Emergency and Disaster Medicine, Wenzhou, 325000, China; ^3^ Cixi Biomedical Research Institute, Wenzhou Medical University, Ningbo, 315300, China, wmu.edu.cn; ^4^ Department of Intensive Care Medicine, Ningbo No. 2 Hospital, Wenzhou Medical University, Ningbo, 315010, Zhejiang, China, wmu.edu.cn; ^5^ Emergency Department, Ningbo No. 2 Hospital, Wenzhou Medical University, Ningbo, 315010, Zhejiang, China, wmu.edu.cn

**Keywords:** group-based trajectory modeling, hospital-acquired bloodstream infections, mortality, sepsis, temperature trajectory

## Abstract

**Purpose:**

Sepsis is a heterogeneous clinical syndrome, and identification of its subphenotypes is essential. We aimed to investigate the correlation between the different trajectories of temperature and outcomes in septic patients.

**Patients and methods:**

This was a retrospective cohort study that included sepsis patients admitted to the intensive care unit (ICU) of a tertiary care hospital. Patients’ temperature data were collected during the first 48 h after ICU admission, and patients’ temperature trajectories were identified using growth‐based trajectory modeling (GBTM). A multivariable logistic regression model was performed to assess the independent association of clinical outcomes. External validation was conducted using the MIMIC‐IV database.

**Results:**

A total of 312 patients with sepsis were included. Four temperature trajectories were identified: hypothermia group (*n* = 33), fever and rapid temperature drop group (*n* = 45), persistently elevated temperature group (*n* = 63), and normothermia group (*n* = 171). Hypothermia patients exhibited the highest mortality and incidence of hospital‐acquired bloodstream infections (HA‐BSIs). Multivariable logistic regression showed that compared with the normothermia group, patients in the hypothermia group (OR 2.73; 95% CI 1.10–6.73; *p* = 0.030) and the persistently elevated temperature group (OR 3.40; 95% CI 1.74–6.67; *p* < 0.001) had significantly higher in‐hospital mortality. Similar subphenotypes were observed in the MIMIC‐IV cohort. Further analysis reached similar results in the MIMIC‐IV dataset.

**Conclusion:**

This study used longitudinal body temperature data to identify the subphenotypes of sepsis, with significant variability in in‐hospital mortality and HA‐BSI. A better understanding of the temperature trajectory may be helpful in identifying deteriorating septic patients in advance.

## 1. Introduction

Sepsis is considered to be due to an overexuberant inflammatory response to infection, leading to substantial morbidity and mortality [1]. Despite decades of large clinical trials, there have been no therapies identified that consistently benefit septic patients overall [2–4]. One of the possible reasons for the negative results of these trials is the issue of the case mix. Therefore, classifying the heterogeneous syndrome of sepsis into distinct “physiological states of interest” may promote the development of precision medicine [5].

Previous studies have identified sepsis subphenotypes using unsupervised methods like latent profile analysis and k‐means clustering analysis, based on vital signs and laboratory measurements [6–8]. Nevertheless, sepsis is a dynamic process where the clinical signs and biomarkers change over minutes to hours [9, 10]. Static indicators may not capture the subphenotypes effectively. Longitudinal data can overcome the limitations of static indicators by capturing disease progression and treatment response. In the intensive care unit (ICU), body temperature in critically ill patients is routinely and frequently monitored. The thermoregulatory response exhibits significant heterogeneity in septic patients. Although fever is commonly regarded as a herald of infection, many septic patients present with normothermia or even hypothermia [11]. Fever and hypothermia are distinct thermoregulatory responses to infection, each having potential adaptive biological value for the host. However, the relationship between temperature and the prognosis of septic patients remains controversial. Several studies showed that fever is recognized to elevate metabolic requirements, minute ventilation, and energy consumption, which could have adverse effects on organ function [12, 13]. Some research observed that the febrile response could promote the immune system and reduce mortality [14, 15]. When the patient encounters a severe homeostatic challenge and the energy required to initiate fever exceeds the potential benefits, hypothermia becomes apparent and tolerant, prompting the host to focus on the conservation of energy and the maintenance of homeostasis [16]. Whether hypothermia serves as a marker for severe infection or an indicator of deteriorating sepsis remains to be explored [11, 17].

Therefore, this study aims to move beyond static, single‐point temperature assessments by classifying septic patients into distinct subphenotypes based on longitudinal temperature trajectories. By capturing the dynamic thermoregulatory patterns, we seek to provide deeper insights into the physiological heterogeneity of sepsis that traditional snapshots may overlook. Furthermore, we aim to examine the relationship between these trajectories and clinical outcomes, providing new evidence for personalized risk stratification and offering clinical insights that may help optimize the timing of monitoring and therapeutic interventions.

## 2. Methods

### 2.1. Study Design and Setting

This was a retrospective cohort study conducted at Ningbo No. 2 Hospital (an urban academic tertiary care hospital). The study was approved by the Ethics Committee of Ningbo No. 2 Hospital (PJ‐NBEY‐KY‐2024‐106‐01), and the requirement for informed consent was waived due to the retrospective nature of the analysis. To ensure the stability of the results, the validation cohort (Medical Information Mart for Intensive Care IV database: MIMIC‐IV) was from Beth Israel Deaconess Medical Center from 2008 to 2019 [18]. To access the database, one of the authors, Keying Ding, obtained the required certification and subsequently extracted the relevant variables for our study (Certification No.: 11801101). Individual patient consent was not required due to the anonymized nature of the patient health information contained within this database. This study was reported according to the Strengthening the Reporting of Observational Studies in Epidemiology (STROBE) guidelines [19].

### 2.2. Participants and Data Collection

Adult patients older than 18 years with sepsis who were admitted to Ningbo No. 2 Hospital (a Class A tertiary general hospital) between January 1, 2020, and December 31, 2023, were included in this study. Sepsis was identified based on the Sepsis‐3 criteria as documented or suspected infection with an acute increase in the Sequential Organ Failure Assessment (SOFA) score of ≥ 2 points. Due to the retrospective nature of the study, prehospital baseline SOFA scores were unavailable for the majority of patients. In accordance with the Sepsis‐3 consensus guidelines, we assumed a baseline SOFA score of zero for all patients without evidence of preexisting organ dysfunction prior to the index admission [20]. Therefore, the first SOFA score of ≥ 2 recorded upon admission was used as a surrogate for an acute increase from baseline. For patients who met these criteria, information on their age, sex, chronic underlying disease status, body mass index (BMI), SOFA score, Acute Physiology and Chronic Health Evaluation II (APACHE II) score, duration of hospitalization, bloodstream infection, use of continuous renal replacement therapy (CRRT), central venous catheterization (CVC), vasopressors, fluid balance, antipyretic use (administered when body temperature exceeded 38.5°C), and laboratory measurements (hemoglobin [Hb], platelet count, white blood cell [WBC] count, total bilirubin [TB], indirect bilirubin [IBIL], albumin, aspartate aminotransferase [AST], alanine aminotransferase [ALT], creatinine, C‐reactive protein [CRP], and procalcitonin [PCT]) was recorded. The laboratory test results were collected within the first 24 h after ICU admission. Severity scores were calculated within the first 24 h after ICU entry. For variables recorded more than once within the first 24 h, we utilized the value associated with the greatest severity of illness. The temperature of the patients (tympanic membrane temperature) was recorded at 4‐h intervals for the first 48 h after admission to the ICU. Patients who stayed in the ICU for fewer than 48 h were excluded. If a patient had multiple ICU admissions, only the first ICU admission was included in the analysis. The primary endpoint of our study was hospital mortality. Hospital‐acquired bloodstream infections (HA‐BSIs) were defined as the isolation of a pathogenic organism from at least one blood culture sampled 48 h or more after hospital admission. Similarly, we extracted data from the MIMIC‐IV database for patients who met the Sepsis 3.0 diagnostic criteria through the execution of a Structured Query Language (SQL), along with the parameters mentioned previously. In the MIMIC‐IV cohort, the Simplified Acute Physiology Score II (SAPS II) was used instead of the APACHE II score.

### 2.3. Data Analysis

Data preprocessing was performed to optimize data quality and reduce potential biases by excluding patients who did not meet the inclusion criteria. Data that satisfied the conditions were cross‐validated via leave‐one‐out cross‐validation (LOOCV), Bayes information criterion (BIC), and Akaike information criterion (AIC) to determine the number of classes. Group‐based trajectory modeling (GBTM) of body temperature was performed with the *R* crimCV package. GBTM, known as latent class growth modeling (LCGM), is a semiparametric modeling method used to identify groups with similar development trajectories among groups. Although each individual has a unique development process, the heterogeneity or distribution among individuals can be reflected by a finite but unique set of polynomial functions. Each polynomial function corresponds to a discrete function trajectory; that is, the relationship between time and latent variables is established through polynomial functions [21]. For normally distributed data, analysis of variance (ANOVA) should be used, while for skewed data, nonparametric tests are more appropriate. The Mann–Whitney *U* test was used to compare the correlations between each trajectory and the clinical characteristics. A multivariable logistic regression model with increasing covariates was used to explore the association between temperature trajectories and mortality. Model 1 was a univariate analysis. Model 2 was adjusted for age and APACHE II. In Model 3, covariate selection was primarily guided by clinical reasoning and expert judgment to ensure the inclusion of all potential confounders, rather than relying solely on automated statistical thresholds. While variables with a univariable *p* value < 0.05 were considered, univariable screening was treated as exploratory and Supporting Information to avoid the omission of clinically significant factors. Furthermore, we implemented inverse probability of treatment weighting (IPTW) to create a weighted cohort, which serves as our primary strategy to minimize baseline imbalances between temperature trajectory groups in the MIMIC‐IV dataset.

For the processing of missing temperature data, backward imputation was performed. There were no missing values for laboratory test results collected in the Ningbo cohort; therefore, no imputation was performed. For the MIMIC‐IV cohort, variables with more than 20% missing data were excluded. For the remaining laboratory parameters, including WBC count, hemoglobin, platelet count, and creatinine, the missing data were considered to be missing at random (MAR), and multiple imputation was applied [22].

The above statistical analysis was performed via *R* (Version 4.3.2) and Stata (Version 17), and a *p* value of less than 0.05 was considered significant.

## 3. Results

**FIGURE 1:**
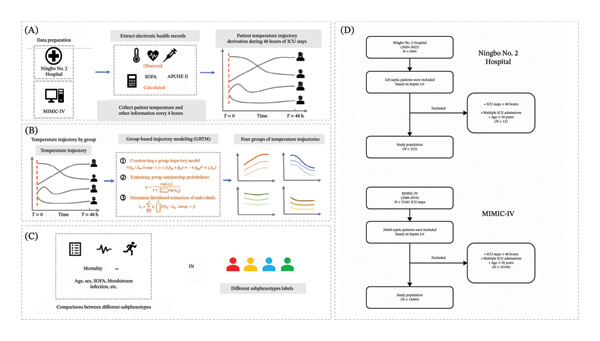
Flowchart of included patients.

**FIGURE 2:**
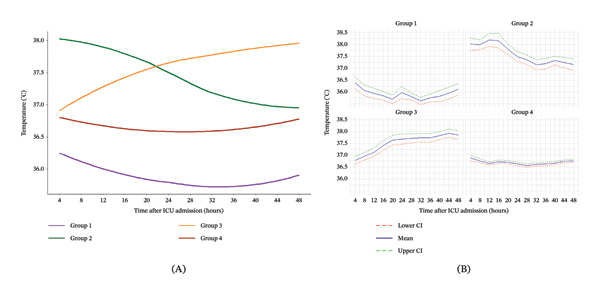
Temperature trajectory in patients with sepsis. Figure 2(A) shows the temperature trajectory of septic patients during the first 48 h after ICU admission. Figure 2(B) presents the 95% confidence intervals for the temperature trajectories. The area between the red and green dashed lines represents the 95% confidence interval, while the blue solid line represents the mean. Group 1, hypothermia group; Group 2, fever and rapid temperature drop group; Group 3, persistently elevated temperature group; Group 4, normothermia group.

A total of 312 patients were enrolled in our study (Figure 1). Of these, 34 temperature data points were missing, accounting for 0.9% of total (3744). Then, the GBTM was fit to the whole dataset. LOOCV, BIC, and AIC analyses were performed to determine the best‐fit model. When the number of classes was 4, the LOOCV, BIC, and AIC values were the lowest. Taken together, the four‐class model was the best. Therefore, the whole septic patients were divided into four groups based on body temperature trajectory: Group 1 (hypothermia group), Group 2 (fever and rapid temperature drop group), Group 3 (persistently elevated temperature group), and Group 4 (normothermia group) (Figure 2). The number of patients in each subphenotype group was 33, 45, 63, and 171, respectively. Meanwhile, a total of 15,469 patients with sepsis were included in the final analysis from the MIMIC‐IV dataset. Similar trajectory trends were obtained with GBTM (Figure S1).

### 3.1. Different Clinical Features Between Groups

The baseline characteristics of each group are described in Table 1 and Table S1. Hypothermia was more prevalent in nonsurvivors than in survivors. Among the four temperature trajectory groups, Group 2 was the youngest with a median age of 68 (*p* = 0.013). Except for a history of kidney disease, the baseline comorbidity rates are significantly similar among different groups. Patients in Group 1 showed the highest SOFA score [12 (IQR 9–15); *p* < 0.001] and creatinine levels [271.70 (IQR 114.50–441.60); *p* < 0.001] and were more likely to use CRRT during the ICU stay (*p* < 0.001). Meanwhile, hemoglobin levels were higher in Group 2 compared to all other cohorts (*p* < 0.001). Group 4 showed less net fluid balance compared to all other groups within the first 24 h after ICU admission [860.80 (IQR −296.50–1787.80); *p* = 0.003]. Similarly, in the MIMIC‐IV cohort, the SOFA score, creatinine levels, and use of CRRT exhibited trends consistent with those previously described (Table S2).

**TABLE 1:** Comparison of patient characteristics within the four temperature trajectory subphenotypes.

	**Group 1**	**Group 2**	**Group 3**	**Group 4**	***p* value**
*N*	33	45	63	171	
Age, yr	74.00 (63.00, 83.00)	68.00 (60.00, 77.00)	72.00 (59.50, 79.00)	75.00 (67.00, 82.00)	**0.013**
Gender, male, *n* (%)	24 (72.73)	23 (51.11)	49 (77.78)	108 (63.16)	**0.023**
BMI, kg/m^2^	22.49 (20.75, 23.88)	23.66 (20.03, 25.95)	23.31 (20.35, 26.56)	21.97 (19.02, 25.30)	0.224
SOFA, s	12 (9, 15)	10 (8, 12)	8 (6, 12)	8 (6, 11)	**< 0.001**
APACHE II, s	27 (20.00, 31.00)	25 (19.00, 30.00)	23 (19.50, 28.50)	23 (18.00, 28.00)	0.150

Comorbidity,					
CHF, *n* (%)	2 (6.06)	1 (2.22)	2 (3.17)	8 (4.68)	0.804
COPD, *n* (%)	2 (6.06)	2 (4.44)	1 (1.59)	12 (7.02)	0.431
Diabetes, *n* (%)	10 (30.30)	13 (28.89)	13 (20.63)	45 (26.32)	0.690
Hypertension, *n* (%)	20 (60.61)	20 (44.44)	28 (44.44)	89 (51.46)	0.397
Liver disease, *n* (%)	1 (3.03)	6 (13.33)	11 (17.46)	34 (19.88)	0.108
Renal disease, *n* (%)	9 (27.27)	3 (6.67)	6 (9.52)	34 (19.88)	**0.023**
Cancer, *n* (%)	9 (27.27)	11 (24.44)	12 (19.05)	41 (23.98)	0.801

*Laboratory tests*					
WBC, 10^9^/L	12.55 (9.00, 17.68)	12.38 (8.54, 17.16)	11.13 (7.56, 16.38)	12.07 (8.41, 16.48)	0.697
Hb, g/L	84.00 (72.00, 99.00)	116.00 (98.00, 127.00)	106.00 (86.50, 133.50)	98.00 (82.00, 118.00)	**< 0.001**
PLT, 10^9^/L	152.00 (80.00, 212.00)	169.00 (118.00, 213.00)	165.00 (93.00, 225.00)	166.00 (105.50, 229.50)	0.737
Total bilirubin, μmol/L	10.40 (6.50, 28.20)	15.80 (8.30, 30.70)	11.30 (7.80, 19.45)	11.00 (7.45, 17.05)	0.172
Indirect bilirubin, μmol/L	5.60 (2.50, 8.70)	6.40 (4.00, 12.30)	6.00 (3.50, 8.40)	5.40 (3.70, 8.60)	0.487
Albumin, g/L	27.40 (25.50, 31.00)	27.40 (23.70, 31.20)	28.80 (23.30, 34.05)	26.70 (23.30, 30.65)	0.380
AST, U/L	44.00 (32.00, 91.00)	57.00 (33.00, 125.00)	59.00 (34.00, 117.00)	46.00 (27.50, 79.00)	0.174
ALT, U/L	19.00 (12.00, 33.00)	26.00 (13.00, 71.00)	34.00 (16.50, 69.50)	24.00 (12.00, 49.50)	0.085
CRP, mg/L	129.15 (75.13, 190.28)	148.25 (81.70, 212.24)	137.88 (86.30, 219.89)	125.38 (74.95, 196.12)	0.587
Creatinine, μmol/L	271.70 (114.50, 441.60)	121.30 (56.90, 213.40)	94.80 (63.40, 139.40)	113.20 (64.40, 219.80)	**< 0.001**
PCT, ng/mL	7.41 (0.90, 15.41)	6.61 (0.85, 33.82)	2.62 (0.91, 24.59)	2.50 (0.38, 13.34)	0.098

*Interventions*					
CRRT, *n* (%) (1st 48 h use)^∗^	16 (48.48)	8 (17.78)	6 (9.52)	25 (14.62)	**< 0.001**
Mechanical ventilation, *n* (%) (1st 48 h use)^∗^	30 (90.91)	41 (91.11)	57 (90.48)	144 (84.21)	0.393
CVC, *n* (%) (1st 48 h use)^∗^	23 (69.70)	32 (71.11)	46 (73.02)	131 (76.61)	0.768
Vasoactive agents, *n* (%) (1st 48 h use)^∗^	23 (69.70)	34 (75.56)	44 (69.84)	112 (65.50)	0.612
Net fluid balance^†^, mL	1616.00 (514.20, 3327.00)	1199.00 (257.40, 2268.00)	1138.20 [264.50, 2662.50]	860.80 (−296.50, 1787.80)	**0.003**
Antipyretic drugs, *n* (%) (1st 48 h use)^∗^	0 (0)	13 (28.89)	13 (20.63)	4 (2.34)	**< 0.001**

*Infection*					
Respiratory, *n* (%)	24 (72.73)	28 (62.22)	35 (55.56)	116 (67.84)	0.251
Gastrointestinal, *n* (%)	3 (9.09)	9 (20)	19 (30.16)	34 (19.88)	0.103
Genitourinary, *n* (%)	2 (6.06)	1 (2.22)	0 (0)	3 (1.75)	0.233
Others, *n* (%)	4 (12.12)	7 (15.56)	9 (14.29)	18 (10.53)	0.758

*Note:* Hb: hemoglobin, PLT: procalcitonin, AST: aspartate aminotransferase, ALT: alanine aminotransferase, CRP: C‐reactive protein, PCT: procalcitonin. All values are expressed as median (interquartile range) or percentages (%). Group 1, hypothermia group; Group 2, fever and rapid temperature drop group; Group 3, persistently elevated temperature group; Group 4, normothermia group. CRRT, mechanical ventilation, central venous catheterization, vasoactive agents, and antipyretic drugs were recorded within 48 h after ICU admission. Bold values indicate statistical significance (*p* < 0.05).

Abbreviations: APACHE II = Acute Physiology and Chronic Health Evaluation II, BMI body mass index, CHF = congestive heart failure, COPD = chronic obstructive pulmonary disease, CRRT = continuous renal replacement therapy, CVC = central venous catheter, SOFA = Sequential Organ Failure Assessment,  = WBC white blood cell.

^†^Net fluid balance was calculated within 24 h after ICU admission.

^∗^The intervention was conducted within 48 h of ICU admission.

### 3.2. Clinical Outcomes

There were significant differences in hospital mortality among the four temperature trajectory groups (*p* < 0.001). Group 1 had the highest mortality rate (66.67%), and Group 4 had the lowest mortality rate (29.82%). The mortality rates of Group 2 (44.44%) and Group 3 (53.97%) fell between those of Groups 1 and 4 (Table 2). By using Group 4 as a reference, Group 1 (OR 4.71; 95% CI 2.13–10.42; *p* < 0.001) and Group 3 (OR 2.76; 95% CI 1.52–5.00; *p* < 0.001) showed increased risks of in‐hospital mortality (Table 3). After adjusting for possible confounders associated with mortality, the association remained significant (Group 1: OR 2.73; 95% CI 1.10–6.73; *p* = 0.030; Group 3: OR 3.40; 95% CI 1.74–6.67; *p* < 0.001) (Table 3 and Table S3). Consistent results were observed in the MIMIC‐IV cohort. Group 1 had the highest mortality rate (28.28%), while Group 3 had the lowest (14.22%) (Table S4). Multivariate logistic regression analysis showed that, compared with Group 2, Group 1 (OR 1.53; 95% CI 1.31–1.78; *p* < 0.001) and Group 4 (OR 1.38; 95% CI 1.15–1.65; *p* < 0.001) were significantly associated with increased in‐hospital mortality (Tables S5 and S6). The standardized mean differences (SMDs) of before and after IPTW are shown in Figure S2. The covariates among the groups were well‐balanced after IPTW. Consistent with the results from the pre‐IPTW model, Group 1 and Group 4 demonstrated increased risks of in‐hospital mortality when using Group 2 as reference (OR 1.60; 95% CI: 1.33–1.93; *p* < 0.001; OR 1.40; 95% CI: 1.11–1.76; *p* = 0.004, Table S7).

**TABLE 2:** Clinical outcomes in the four temperature trajectory subphenotypes.

	**Group 1 (*N* = 33)**	**Group 2 (*N* = 45)**	**Group 3 (*N* = 63)**	**Group 4 (*N* = 171)**	***p* value**

Length of hospital stay, d	19.00 (9.00, 38.00)	12.00 (7.00, 23.00)	16.00 (6.00, 28.00)	15.00 (9.00, 23.00)	0.368
Hospital mortality *n* (%)	22 (66.67%)	20 (44.44%)	34 (53.97%)	51 (29.82%)	< 0.001
HA‐BSI, *n* (%)	9 (27.27%)	5 (11.11%)	2 (3.17%)	14 (8.19%)	0.002

*Note:* Group 1, hypothermia group; Group 2, fever and rapid temperature drop group; Group 3, persistently elevated temperature group; Group 4, normothermia group.

Abbreviations: HA‐BSI = hospital‐acquired bloodstream infections.

**TABLE 3:** Association between hospital mortality and temperature trajectory subphenotypes in logistic regressions.

	**Model 1**		**Model 2**		**Model 3**	
	**OR (95% CI)**	***p* Value**	**OR (95% CI)**	***p* Value**	**OR (95% CI)**	***p* value**

Group 4	Ref.		Ref.		Ref.	
Group 1	4.71 (2.13, 10.42)	< 0.001	4.32 (1.86, 10.00)	< 0.001	2.73 (1.10, 6.73)	0.030
Group 2	1.88 (0.96, 3.69)	0.066	1.85 (0.90, 3.81)	0.096	1.75 (0.84, 3.67)	0.137
Group 3	2.76 (1.52, 5.00)	< 0.001	3.09 (1.63, 5.85)	< 0.001	3.40 (1.74, 6.67)	< 0.001

*Note:* Model 1 was an univariate analysis without adjusting any covariates; Model 2 was adjusted for age and APACHE II; Model 3 was adjusted for age, SOFA, APACHE II, PLT, creatinine, PCT, CRRT, mechanical ventilation, and vasopressor use. Group 1, hypothermia group; Group 2, fever and rapid temperature drop group; Group 3, persistently elevated temperature group; Group 4, normothermia group.

Abbreviation: OR = Odds Ratio.

HA‐BSI rates varied across different subphenotypes (*p* = 0.002), with Group 1 having the highest incidence of HA‐BSI (27.27%) and Group 3 showing the lowest incidence (3.17%) (Table 2). No statistically significant difference was observed in the duration of hospital stay between the groups in the Ningbo cohort.

## 4. Discussion

In this study, we identified four temperature trajectories of sepsis: Group 1 was characterized by hypothermia, Group 2 was characterized by fever and rapid temperature drop, Group 3 was characterized by persistently elevated temperature, and Group 4 was characterized by normothermia. The four subphenotypes of sepsis had different clinical features and outcomes. These findings could play a role in a precision medicine–based approach to sepsis management.

Recent studies have demonstrated that identifying biologically meaningful classes of a disease could facilitate more personalized management strategies. Several research studies have concentrated on identifying subgroups of sepsis based on genomic and transcriptomic data [23–25]. Davenport et al. identified two distinct subtypes of sepsis caused by community‐acquired pneumonia based on global gene expression, one of which showed immunosuppression and was associated with increased mortality [23]. Yang et al. identified two distinct subgroups of septic shock in pediatric patients by analyzing whole‐blood RNA expression profiles. Remarkably, these two subclasses exhibit differential regulation of immune system–related genes that are pertinent to the pathophysiology of sepsis and septic shock [24]. However, transcriptomic subtyping is expensive and not commonly utilized in real clinical settings, which restricts its broad application. Comprehensive electronic health records (EHRs) can reveal the inherent heterogeneity of critical illness. In this context, bedside measurements offer a practical method to identify sepsis subphenotypes.

Recently, Thomas‐Rüddel et al. reported a bimodal distribution of body temperature among septic patients, and hypothermia was associated with elevated 28‐day mortality [26]. This study recorded the most pathological temperature within the first day after sepsis onset. In real clinical settings, the temperature of each critically ill patient is assessed regularly and repeatedly. Our study used a repository of longitudinal quantitative data provided by septic patients’ temperature data. With the GBTM method, we were able to summarize individual differences of septic patients in the developmental trajectory of a clinical variable more comprehensively. Similar to our study, Bhavani and colleagues identified four temperature trajectory groups based on measurements within the first 72 h of septic patient admissions through a series of studies: “hyperthermic, slow resolvers,” “hyperthermic, fast resolvers,” “normothermic,” and “hypothermic” [27, 28]. Broadly mirroring our observations, in patients with sepsis, hypothermia was more frequently observed in nonsurvivors and was associated with increased mortality compared with normothermia [27]. Our fever and rapid temperature drop subgroup corresponded to the “hyperthermic, fast resolvers” group; our persistently elevated temperature group likely corresponded to the “hyperthermic, slow resolvers” group. Body temperature trajectories may represent an important clinical phenotype reflecting the dynamic immune response of the host during sepsis. Hypothermia has been associated with immune suppression and worse outcomes, whereas fever may reflect an active host defense response against infection. Distinct temperature trajectory subphenotypes likely represent different stages or patterns of immune response, highlighting the immunological heterogeneity of sepsis and potentially guiding individualized therapeutic strategies [27, 28]. Sepsis typically has a rapid onset and is associated with a high short‐term mortality [29, 30]. Given the critical window for intervention in sepsis, early recognition of subphenotypes of sepsis is crucial. In our study, we constructed trajectory models using temperature measurements taken within 48 h of ICU admission. These findings not only confirm the distinct temperature trajectories in septic patients but also minimize the potential bias arising from early mortality (within 3 days) due to severe disease, thus better aligning with real‐world clinical practice.

Qi et al. showed that hemoglobin levels ≤ 80 g/L may be a useful predictor of mortality for septic patients [31]. In our study, the hypothermia group had the lowest hemoglobin levels, whereas the fever and rapid temperature drop group had the highest. Further large‐scale research is needed to confirm these associations.

Another important finding of our study is that many septic patients with HA‐BSI rarely have a fever. It is notable that Benzoni et al. reported a certain incidence of BSIs in the normothermia and hypothermia group of oncology patients with neutropenia and suspected infection [32]. Our findings served as an additional validation of BSIs, indicating that septic patients without high fever also warrant significant attention due to their risk of HA‐BSI, particularly those with hypothermia.

Several limitations must be acknowledged in the present study. Firstly, using an absolute admission SOFA score instead of a change from baseline might slightly overestimate sepsis incidence among patients with chronic organ dysfunction, a common constraint in retrospective studies where baseline data are often missing. Additionally, the retrospective design carries inherent risks of selection bias and residual confounding, which we addressed using IPTW but could not completely exclude. Moreover, although our results were externally validated with the MIMIC database, they should be interpreted with caution until confirmed by larger, prospective observational trials. Secondly, although our study presents single‐variable trajectories, we acknowledge the potential benefits of integrating multiple variables into a composite panel. Combining temperature with other dynamic parameters could yield more comprehensive clinical insights. Further large‐scale and well‐designed studies would be useful to provide more evidence. Thirdly, our findings delineate clinical trajectories but do not establish any association between specific interventions and their effectiveness regarding these outcomes. Further research is needed to determine whether specific interventions offer survival benefits for patients with distinct temperature trajectories. Fourthly, the study population consisted exclusively of ICU patients, representing a more severely ill subgroup of sepsis patients, which may limit the generalizability of our findings to patients treated outside the ICU. Fifthly, temperature was measured via tympanic thermometry, a common but less precise surrogate for core temperature than invasive methods. Factors like probe positioning and environmental interference might introduce measurement noise. However, this represents the most practical data source available in our retrospective cohort. Future prospective studies with more precise monitoring are warranted to validate the stability of these temperature subphenotypes. Finally, it is difficult to draw a causal association by observational design. However, external validation and IPTW analysis showed consistent results, which enhanced the robustness of the findings. The underlying mechanisms of the temperature changes in patients with sepsis deserve further investigation.

## 5. Conclusion

Our study has identified four temperature trajectories of body temperature among septic patients with different clinical characteristics and outcomes, which reflect the dynamic complexity of sepsis. These findings may facilitate the transition from a one‐size‐fits‐all approach to precision medicine in sepsis management.

**Nomenclature**
ICU: intensive care unit
GBTM: growth‐based trajectory modeling
HA‐BSI: hospital‐acquired bloodstream infections
MIMIC‐IV: Medical Information Mart for Intensive Care IV
STROBE: Strengthening the Reporting of Observational Studies in Epidemiology
SOFA: Sequential Organ Failure Assessment
BMI: body mass index
APACHE II: Acute Physiology and Chronic Health Evaluation II
CRRT: continuous renal replacement therapy
CVC: central venous catheterization
WBC: white blood cell
TB: total bilirubin
IBIL: indirect bilirubin
AST: aspartate aminotransferase
ALT: alanine aminotransferase
CRP: C‐reactive protein
PCT: procalcitonin
SQL: Structured Query Language
SAPS II: Simplified Acute Physiology Score II
LOOCV: leave‐one‐out cross‐validation
BIC: Bayes information criterion
AIC: Akaike information criterion
LCGM: latent class growth modeling
ANOVA: analysis of variance
IPTW: inverse probability of treatment weighting
MAR: missing at random
EHRs: electronic health records

## Author Contributions

Keying Ding: writing–original draft, investigation, data curation, methodology, and software. Chang Xu: writing–original draft, conceptualization, methodology, and formal analysis. Xueqi Zhu: data curation, formal analysis, investigation, and validation. Hua Wang: writing–review and editing, validation, and visualization. Zhaojun Xu: writing–review and editing, supervision, and validation. Zhongqiu Lu: writing–review and editing, supervision, conceptualization, and project administration.

## Funding

This work was supported by Zhejiang Province Clinical Key Specialized Emergency Medicine Department and Zhejiang Provincial Interdisciplinary Traditional Chinese Medicine Innovation Team for Diagnosis and Treatment of Sepsis.

## Ethics Statement

This study was approved by the ethics committee of Ningbo No. 2 Hospital (PJ‐NBEY‐KY‐2024‐106‐01). The study conformed to the provisions of the Declaration of Helsinki (as revised in 2013).

## Conflicts of Interest

The authors declare no conflicts of interest.

## Supporting Information

Additional supporting information can be found online in the Supporting Information section.

## Supporting information


**Supporting Information** Additional supporting information can be found online in the Supporting Information section. Supporting Information Table S1: Comparison of temperature parameters (Ningbo cohort). Supporting Information Table S2: Baseline characteristics of patients across the four temperature trajectory subphenotypes (MIMIC‐IV). Supporting Information Table S3: Potential risk variables for impact on prognosis (Ningbo cohort). Supporting Information Table S4: Clinical outcomes (MIMIC‐IV). Supporting Information Table S5: Association between hospital mortality and temperature trajectory subphenotypes in logistic regressions (MIMIC‐IV). Supporting Information Table S6: Potential risk variables for impact on prognosis (MIMIC‐IV). Supporting Information Table S7: Association between hospital mortality and temperature trajectory subphenotypes in logistic regressions (MIMIC‐IV‐IPTW). Supporting Information Figure S1: Temperature trajectory (MIMIC‐IV). Supporting Information Figure S2: Standard mean differences in covariates before and after IPTW.

## Data Availability

The dataset used during the current study is available from the corresponding authors on reasonable request.
