# Supplementary material for: Temperature Trajectories Are Associated With the Prognosis of Septic Patients
Source: Emerg Med Int. 2026 May 30;2026:9111659. doi: 10.1155/emmi/9111659 (PMC13239043; doi:10.1155/emmi/9111659)
Supplement: Supplementary file 1 — Supporting Information Additional supporting information can be found online in the Supporting Information section. Supporting Information Table S1: Comparison of temperature parameters (Ningbo cohort). Supporting Information Table S2: Baseline characteristics of patients across the four temperature trajectory subphenotypes (MIMIC‐IV). Supporting Information Table S3: Potential risk variables for impact on prognosis (Ningbo cohort). Supporting Information Table S4: Clinical outcomes (MIMIC‐IV). Supporting Information Table S5: Association between hospital mortality and temperature trajectory subphenotypes in logistic regressions (MIMIC‐IV). Supporting Information Table S6: Potential risk variables for impact on prognosis (MIMIC‐IV). Supporting Information Table S7: Association between hospital mortality and temperature trajectory subphenotypes in logistic regressions (MIMIC‐IV‐IPTW). Supporting Information Figure S1: Temperature trajectory (MIMIC‐IV). Supporting Information Figure S2: Standard mean differences in covariates before and after IPTW. [file EMMI-2026-9111659-s001.docx]

**Supplementary material**

Table S1 Comparison of temperature parameters by survival status

| Temperature parameters | Survivors  (n = 185) | Nonsurvivors  (n = 127) | *P* value |
| --- | --- | --- | --- |
| Presenting temperature, °C | 36.80 (36.40-37.40) | 36.80 (36.45-37.35) | 0.874 |
| Temperature_mean_ | 36.79 (36.56-37.13) | 36.88 (36.40-37.45) | 0.761 |
| Temperature_min_ | 36.10 (35.90-36.40) | 36.00 (35.50-36.40) | 0.197 |
| Temperature_max_ | 37.60 (37.10-38.20) | 38.10 (37.10-38.90) | 0.016 |
| Temperature ≥ 37.5°C, n (%) | 101 (54.59%) | 78 (61.42%) | 0.231 |
| Temperature < 36.0°C, n (%) | 47 (25.41%) | 48 (37.80%) | 0.019 |

Table S2. Comparison of patient characteristics within the four temperature trajectory subphenotypes (MIMIC-IV)

|  | Group 1 | Group 2 | Group 3 | Group 4 | *P* value |
| --- | --- | --- | --- | --- | --- |
| N | 1188 | 8249 | 4851 | 1181 |  |
| **Age, yr** | 73.01  [61.13, 82.94] | 70.67  [60.44, 80.78] | 64.57  [53.07,75.27] | 58.29  [45.68, 69.29] | **< 0.001** |
| **Gender, Male, n (%)** | 643 (54.12) | 4561 (55.29) | 2772 (57.14) | 746 (63.17) | **< 0.001** |
| **Race,White, n(%)** | 841 (70.79) | 5784 (70.12) | 3082 (63.53) | 717 (60.71) | **< 0.001** |
| **SOFA, s** | 7  [4, 10] | 6  [4, 8] | 6  [4, 8] | 6  [4, 9] | **< 0.001** |
| **SAPS II, s** | 46  [38, 56] | 41  [33, 50] | 38  [30, 48] | 40  [31, 49] | **< 0.001** |
| Comorbidity, |  |  |  |  |  |
| **CHF, n (%)** | 519 (43.69) | 3151 (38.20) | 1396 (28.78) | 271 (22.95) | **< 0.001** |
| **COPD, n (%)** | 392 (33.00) | 2616 (31.71) | 1318 (27.17) | 274 (23.20) | **< 0.001** |
| Diabetes, n (%) | 392 (33.00) | 2814 (34.11) | 1576 (32.49) | 379 (32.09) | 0.196 |
| Hypertension, n (%) | 438 (36.87) | 3201 (38.80) | 1918 (39.54) | 473 (40.05) | 0.316 |
| **Liver disease, n (%)** | 292 (24.58) | 1431 (17.35) | 742 (15.30) | 198 (16.77) | **< 0.001** |
| **Renal disease, n (%)** | 451 (37.96) | 2457 (29.79) | 1019 (21.01) | 170 (14.39) | **< 0.001** |
| **Cancer, n (%)** | 187 (15.74) | 1506 (18.26) | 687 (14.16) | 140 (11.85) | **< 0.001** |
| Laboratory tests |  |  |  |  |  |
| **WBC, 10^9/L** | 10.50  [7.20, 15.90] | 11.60  [8.00, 16.20] | 11.80  [8.30, 16.40] | 12.40  [8.40, 16.80] | **< 0.001** |
| **Hb, g/L** | 99.00 | 100.00 | 105.00 | 109.00 | **< 0.001** |
|  | [86.00, 112.00] | [85.00, 115.00] | [90.00, 119.00] | [94.00, 126.00] |  |
| **PLT, 10^9/L** | 178.00  [112.75, 258.25] | 185.00  [126.00, 257.00] | 191.00  [134.00, 262.00] | 191.00  [136.00, 256.00] | **< 0.001** |
| **Creatinine, μmol/L** | 114.92  [70.72, 203.32] | 97.24  [70.72, 167.96] | 97.24  [61.88, 150.28] | 88.40  [70.72, 141.44] | **< 0.001** |
| Interventions |  |  |  |  |  |
| **CRRT, n (%)**  **(1st 48 use)** | 135 (11.36) | 313 (3.79) | 115 (2.37) | 24 (2.03) | **< 0.001** |
| **Mechanical ventilation, n (%)**  **(1st 48 use)** | 615 (51.77) | 4226 (51.23) | 3439 (70.89) | 998 (84.50) | **< 0.001** |
| **Vasoactive agents, n (%) (1st 48 use)** | 645 (54.29) | 4153 (50.35) | 2352 (48.48) | 624 (52.83) | **0.003** |
| **Net fluid balance, ml** | 2710.25  [526.63,5695.13] | 2360.27  [441.00, 5116.00] | 2587.75  [589.59, 5170.38] | 2728.62  [941.77,5726.85] | **< 0.001** |
| **Antipyretic drugs, n (%) (1st 48 use)** | 218 (18.35) | 1690 (20.49) | 1129 (23.27) | 340 (28.78) | **< 0.001** |
| Antibiotic drugs, n (%) (1st 48 use) | 1081(90.99) | 7562(91.67) | 4409(90.89) | 1065(90.18) | 0.218 |
| Infection |  |  |  |  |  |
| **Respiratory, n (%)** | 292 (24.58) | 1961 (23.77) | 1306 (26.92) | 404 (34.21) | **< 0.001** |
| **Gastrointestinal, n (%)** | 146 (12.29) | 671 (8.13) | 336 (6.93) | 96 (8.13) | **< 0.001** |
| **Genitourinary, n (%)** | 190 (15.99) | 1185 (14.37) | 643 (13.25) | 118 (9.99) | **< 0.001** |
| **Others, n (%)** | 560 (47.14) | 4432 (53.73) | 2566 (52.89) | 563 (47.67) | **< 0.001** |

**Abbreviations:** *SOFA* Sequential Organ Failure Assessment, *SAPSII* Simplified Acute Physiology Score Ⅱ, *CHF* congestive heart failure, *COPD* chronic obstructive pulmonary disease, *WBC* white blood cell, *Hb* hemoglobin, *PLT* procalcitonin, *CRRT* continuous renal replacement therapy.

All values are expressed as median (interquartile range) or percentages (%).

CRRT, mechanical ventilation, central venous catheterization, vasoactive agents, antipyretic drugs and antibiotic drugs were recorded within 48 hours after ICU admission. Net fluid balance was calculated within 24 hours after ICU admission.

Table S3. Potential risk variables for impact on prognosis (Ningbo cohort).

|  | OR | 95% CI | *P* value |
| --- | --- | --- | --- |
| Age, yr | 1.01 | 0.99-1.03 | 0.205 |
| Gender, Male, n (%) | 1.34 | 0.83-2.17 | 0.230 |
| BMI, kg/m² | 0.99 | 0.94-1.04 | 0.702 |
| SOFA, s | 1.20 | 1.12-1.28 | < 0.001 |
| APACHE II, s | 1.09 | 1.06-1.13 | < 0.001 |
| Comorbidity, |  |  |  |
| CHF, n (%) | 0.42 | 0.11-1.57 | 0.199 |
| COPD, n (%) | 0.78 | 0.28-2.18 | 0.641 |
| Diabetes, n (%) | 1.51 | 0.91-2.51 | 0.114 |
| Hypertension, n (%) | 1.14 | 0.73-1.79 | 0.565 |
| Liver disease, n (%) | 0.98 | 0.54-1.81 | 0.959 |
| Renal disease, n (%) | 1.43 | 0.79-2.61 | 0.237 |
| Cancer, n (%) | 1.02 | 0.60-1.74 | 0.938 |
| Laboratory tests |  |  |  |
| WBC, 10^9/L | 0.99 | 0.97-1.02 | 0.714 |
| Hb, g/L | 1.00 | 0.99-1.01 | 0.582 |
| PLT, 10^9/L | 1.00 | 0.99-1.00 | 0.008 |
| Total bilirubin, μmol/L | 1.00 | 1.00-1.01 | 0.284 |
| Indirect bilirubin, μmol/L | 1.00 | 0.98-1.02 | 0.859 |
| Albumin, g/L | 1.00 | 0.97-1.04 | 0.808 |
| AST, U/L | 1.00 | 1.00-1.00 | 0.180 |
| ALT, U/L | 1.00 | 1.00-1.00 | 0.613 |
| CRP, mg/L | 1.00 | 1.00-1.00 | 0.966 |
| Creatinine, μmol/L | 1.00 | 1.00-1.00 | < 0.001 |
| PCT, ng/ml | 1.01 | 1.00-1.02 | 0.045 |
| Interventions |  |  |  |
| CRRT, n (%) | 2.37 | 1.31-4.29 | 0.004 |
| Mechanical ventilation, n (%) | 2.64 | 1.21-5.76 | 0.015 |
| Central venous catheterization, n (%) | 1.20 | 0.71-2.02 | 0.499 |
| Vasoactive agents, n (%) | 2.06 | 1.24-3.42 | 0.006 |
| Net fluid balance, ml | 1.00 | 1.00-1.00 | 0.058 |
| Antipyretic drugs, n (%) | 1.96 | 0.82-4.94 | 0.758 |
| Infection |  |  |  |
| Respiratory | 0.72 | 0.45-1.15 | 0.172 |
| Gastrointestinal | 1.12 | 0.65-1.95 | 0.684 |
| Genitourinary | 0.46 | 0.06-2.40 | 0.377 |
| Others | 1.95 | 0.97-4.08 | 0.065 |

**Abbreviations**: *SOFA* Sequential Organ Failure Assessment, *APACHE II* Acute Physiology and Chronic Health Evaluation-II, *BMI* Body mass index, *WBC* white blood cell, *Hb* hemoglobin, *PLT* platelets, *AST* aspartate aminotransferase, *ALT* alanine aminotransferase, *CRP* C-reactive protein, *PCT* procalcitonin, *CRRT* continuous renal replacement therapy. *OR* Odds Ratio, *CI* Confidence Interval.

CRRT, mechanical ventilation, central venous catheterization, vasoactive agents, and antipyretic drugs was recorded within 48 hours after ICU admission. Net fluid balance was calculated within 24 hours after ICU admission.

Table S4. Clinical outcomes in the four temperature trajectory subphenotypes (MIMIC-IV)

|  | Group 1  (N=1188) | Group 2  (N=8249) | Group 3  (N=4851) | Group 4  (N=1181) | *P* value |
| --- | --- | --- | --- | --- | --- |
| Length of hospital stay, d | 9.63 [6.01,16.27] | 9.69 [6.21,15.97] | 10.85 [6.88, 18.20] | 14.01 [8.82, 22.74] | < 0.001 |
| Hospital mortality, n (%) | 336 (28.28%) | 1379 (16.72%) | 690 (14.22%) | 221 (18.71%) | < 0.001 |

Table S5. Association between hospital mortality and temperature trajectory subphenotypes in logistic regressions (MIMIC-IV)

|  | Model 1 | | Model 2 | | Model 3 | |
| --- | --- | --- | --- | --- | --- | --- |
|  | OR (95% CI) | *P* value | OR (95% CI) | *P* value | OR (95% CI) | *P* value |
| Group 2 | Ref. |  | Ref. |  | Ref. |  |
| Group 1 | 1.96 (1.71, 2.26) | < 0.001 | 1.57 (1.36, 1.82) | < 0.001 | 1.53 (1.31, 1.78) | < 0.001 |
| Group 3 | 0.83 (0.75, 0.91) | < 0.001 | 0.91 (0.82, 1.01) | 0.073 | 0.95 (0.85, 1.06) | 0.371 |
| Group 4 | 1.15 (0.98, 1.34) | 0.088 | 1.27 (1.07, 1.50) | < 0.001 | 1.38 (1.15, 1.65) | < 0.001 |

Abbreviations: *OR* Odds Ratio

Model 1 was univariate analysis without adjusting any covariates; Model 2 was adjusted for age and SAPS II; Model 3 was adjusted for age, SOFA, SAPS II, Cancer, COPD, Liver disease, CHF, Renal disease, Hemoglobin, WBC, race, Antipyretic drugs, PLT, CRRT, mechanical ventilation, vasopressor use, respiratory infection, gastrointestinal infection, genitourinary infection, others infection.

Table S6. Potential risk variables for impact on prognosis (MIMIC-IV)

|  | OR | 95% CI | *P* value |
| --- | --- | --- | --- |
| Age, yr | 1.02 | 1.02-1.02 | < 0.001 |
| Gender, Male, n (%) | 0.94 | 0.87-1.02 | 0.159 |
| Race,White,n(%) | 0.83 | 0.76-0.90 | < 0.001 |
| SOFA, s | 1.18 | 1.17-1.19 | < 0.001 |
| SAPS II, s | 1.05 | 1.05-1.05 | < 0.001 |
| Comorbidity, |  |  |  |
| CHF, n (%) | 1.41 | 1.29-1.53 | < 0.001 |
| COPD, n (%) | 1.12 | 1.02-1.23 | 0.013 |
| Diabetes, n (%) | 0.93 | 0.85-1.01 | 0.101 |
| Hypertension, n (%) | 0.93 | 0.85-1.01 | 0.090 |
| Liver disease, n (%) | 2.08 | 1.88-2.29 | < 0.001 |
| Renal disease, n (%) | 1.35 | 1.23-1.48 | < 0.001 |
| Cancer, n (%) | 2.22 | 2.01-2.45 | < 0.001 |
| Laboratory tests |  |  |  |
| WBC, 10^9/L | 1.01 | 1.00-1.01 | < 0.001 |
| Hb, g/L | 0.94 | 0.92-0.96 | < 0.001 |
| PLT, 10^9/L | 1.00 | 1.00-1.00 | < 0.001 |
| Creatinine, μmol/L | 1.02 | 1.00-1.04 | 0.070 |
| Interventions |  |  |  |
| CRRT, n (%) | 2.77 | 2.21-3.45 | < 0.001 |
| Mechanical ventilation, n (%) | 1.31 | 1.09-1.57 | < 0.001 |
| Vasoactive agents, n (%) | 1.53 | 1.41-1.67 | < 0.001 |
| Net fluid balance, ml | 1.00 | 1.00-1.00 | 0.103 |
| Antipyretic drugs, n (%) | 0.83 | 0.75-0.92 | < 0.001 |
| Antibiotic drugs, n (%) | 0.93 | 0.89-1.21 | 0.053 |
| Infection |  |  |  |
| Respiratory | 1.74 | 1.59-1.90 | < 0.001 |
| Gastrointestinal | 1.31 | 1.13-1.51 | < 0.001 |
| Genitourinary | 0.83 | 0.73-0.94 | < 0.001 |
| Others | 0.63 | 0.58-0.69 | < 0.001 |

**Abbreviations**: *SOFA* Sequential Organ Failure Assessment, *SAPSII* Simplified Acute Physiology Score Ⅱ, *WBC* white blood cell, *Hb* hemoglobin, *PLT* platelets, *CRRT* continuous renal replacement therapy. *OR* Odds Ratio, *CI* Confidence Interval.

CRRT, mechanical ventilation, central venous catheterization, vasoactive agents, antipyretic drugs and antibiotic drugs were recorded within 48 hours after ICU admission. Net fluid balance was calculated within 24 hours after ICU admission.

Table S7. Association between hospital mortality and temperature trajectory subphenotypes in logistic regressions (MIMIC IV-IPTW)

|  | Model 1 | | Model 2 | |
| --- | --- | --- | --- | --- |
|  | OR (95% CI) | *P* value | OR (95% CI) | *P* value |
| Group 2 | Ref. |  | Ref. |  |
| Group 1 | 1.58 (1.33, 1.88) | < 0.001 | 1.60 (1.33, 1.93) | < 0.001 |
| Group 3 | 0.95 (0.86, 1.06) | 0.336 | 0.93 (0.83, 1.04) | 0.209 |
| Group 4 | 1.38 (1.11, 1.70) | 0.003 | 1.40 (1.11, 1.76) | 0.004 |

Abbreviations: *OR* Odds Ratio

Model 1 was univariate analysis without adjusting any covariates; Model 2 was adjusted for SOFA, SAPS II, Cancer, COPD, Liver disease, CHF, Renal disease, Hemoglobin, WBC, race, Antipyretic drugs, PLT, CRRT, mechanical ventilation, vasopressor use, respiratory infection, gastrointestinal infection, genitourinary infection, others infection.

**
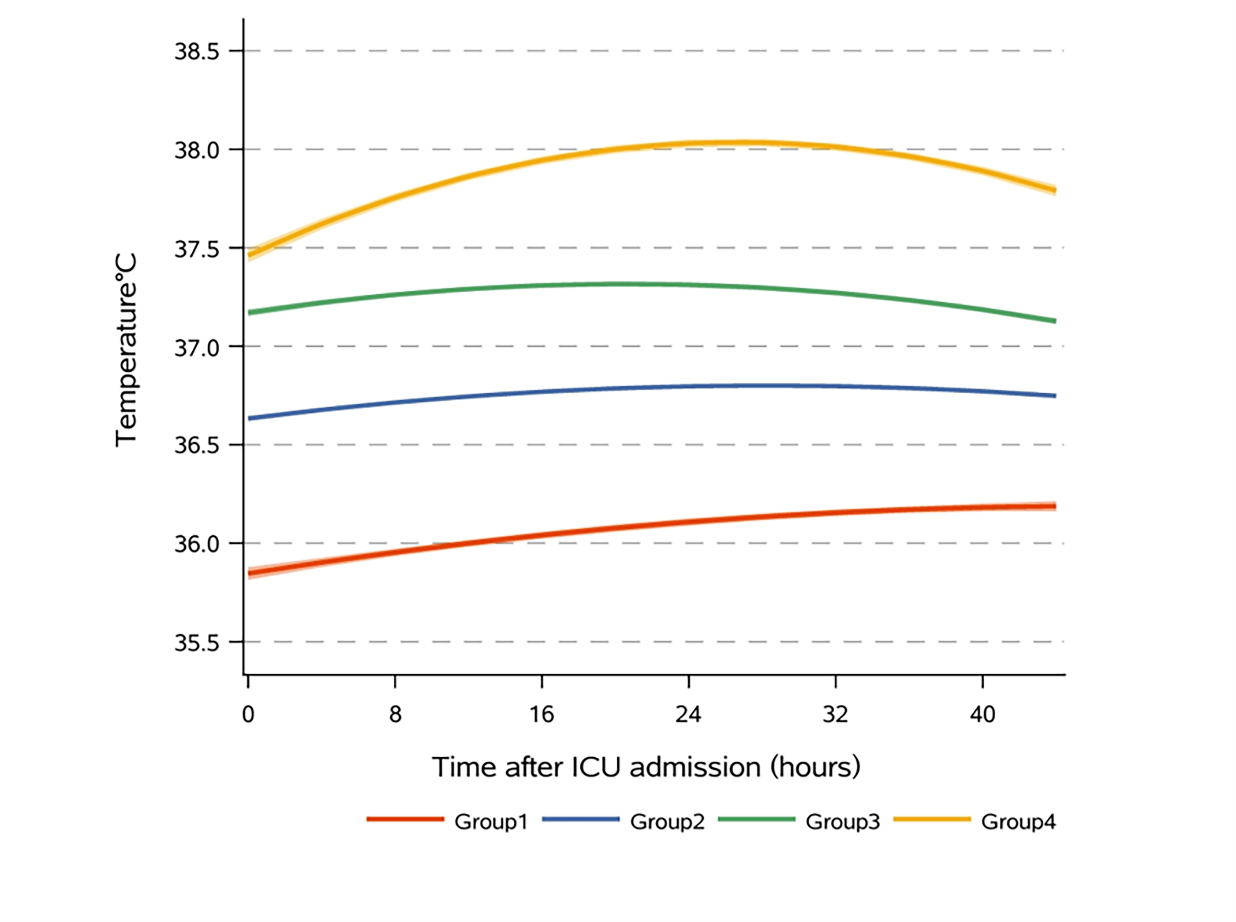
**

**Figure S1**. Temperature trajectory in patients with sepsis (MIMIC-IV).

Group 1, hypothermia group; Group 2, normothermia group; Group 3, fever and temperature drop group; Group 4, persistently elevated temperature group.


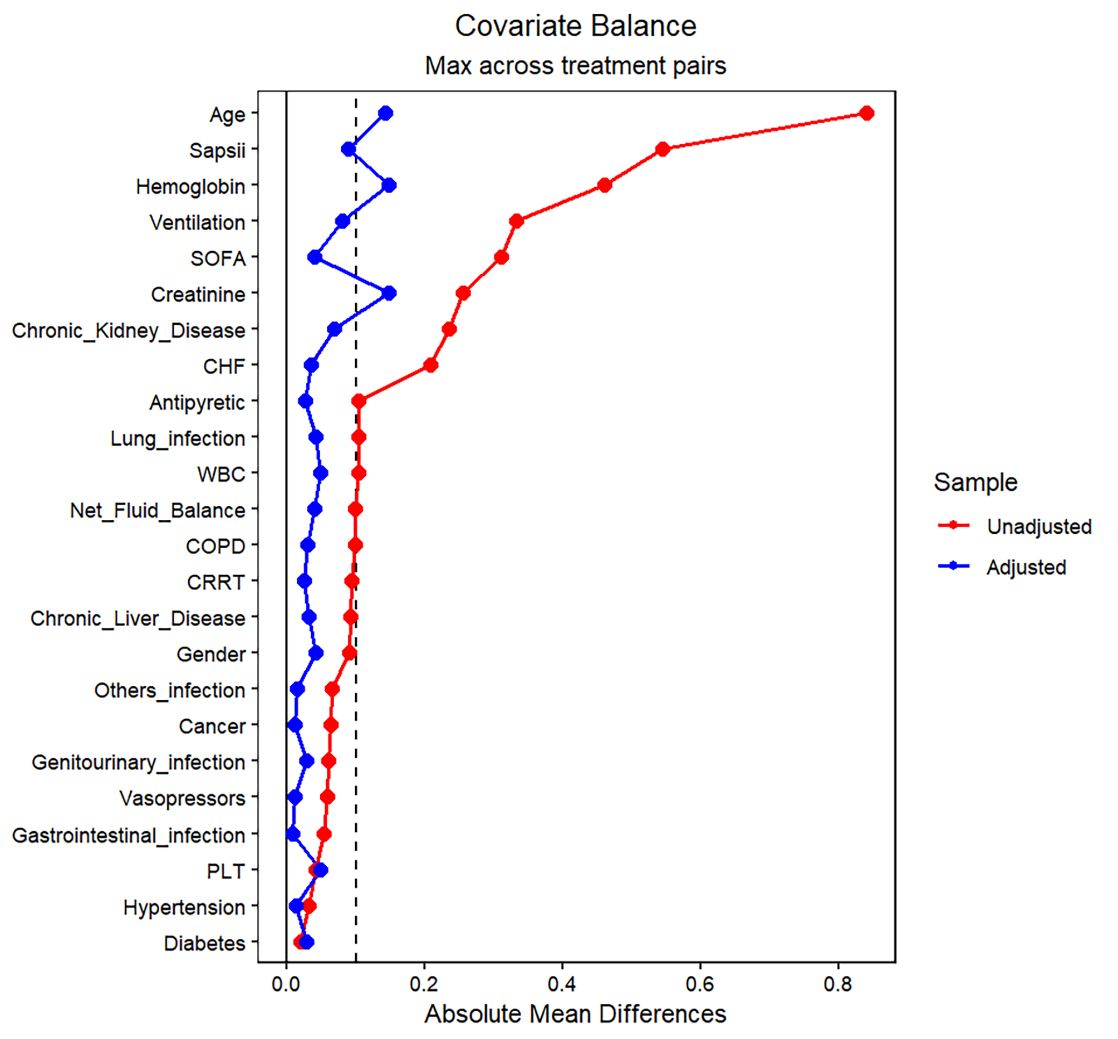


**Figure S2**. Standard mean differences in covariates before and after IPTW.
